# Determinants of AIDS and non-AIDS related mortality among people living with HIV in Shiraz, southern Iran: a 20-year retrospective follow-up study

**DOI:** 10.1186/s12879-019-4676-x

**Published:** 2019-12-30

**Authors:** Zahra Gheibi, Zahra Shayan, Hassan Joulaei, Mohammad Fararouei, Shohreh Beheshti, Mostafa Shokoohi

**Affiliations:** 1 Department of Biostatistics, School of Medicine, Shiraz University of Medical Sciences, Shiraz, Iran; 2 Trauma Research Center, Department of Biostatistics, School of Medicine, Shiraz University of Medical Sciences, Shiraz, Iran; 3 Health Policy Research Center, Institute of Health, Shiraz University of Medical Sciences, Shiraz, Iran; 4 Department of Epidemiology, School of Health & Nutrition, Shiraz University of Medical Sciences, Shiraz, Iran; 5 HIV/AIDS Research Center, Institute of Health, Shiraz University of Medical Sciences, Shiraz, Iran; 6 HIV/STI Surveillance Research Center and WHO Collaborating Center for HIV Surveillance Institute for Future Studies in Health, Kerman University of Medical Sciences, Kerman, Iran; 7 Division of Social and Behavioral Health Sciences, Dalla Lana School of Public Health, University of Toronto, Toronto, ON Canada

**Keywords:** HIV, AIDS, Risk factors, Death, Competing risk

## Abstract

**Background:**

Human Immunodeficiency Virus (HIV) infection has become a global concern. Determining the factors leading to death among HIV patients helps controlling Acquired Immune Deficiency Syndrome (AIDS) epidemic. Up to now, little is known about mortality and its determinants among people living with HIV in the Middle East and North Africa (MENA) region, including Iran. The purpose of this study was to assess the risk factors of AIDS-Related Mortality (ARM) and Non-AIDS-Related Mortality (NARM) among people with HIV in Iran.

**Methods:**

This 20-year retrospective study was conducted on 1160 people with HIV whose data were collected from 1997 to 2017. The association of the study outcomes (ARM and NARM) with various study variables, including demographic status at the time of diagnosis and clinical indexes during the follow-up were examined to define the predictors of mortality among the patients. Regarding, Cox proportional hazard and competing risk models were fitted and Adjusted Hazard Ratios (AHR), Sub-distribution Hazard Ratio (SHR) and the 95% Confidence Intervals (CI) were reported.

**Results:**

during the follow-up period, 391 individuals (33.7%) died with 86,375 person-years of follow-up. Of the total deaths, 251 (64.2%) and 140 (35.8%) were ARM and NARM, respectively. Rates of the mortality caused by AIDS and non-AIDS were 3.2 and 4.5 per 1000 person-months, respectively. Responding to combined Antiretroviral Treatment (cART) 6 months after initiation, receiving Pneumocystis Pneumonia (PCP) prophylaxis, and higher CD4 count at diagnosis, reduced the hazard of ARM and NARM. However, older age, late HIV diagnosis, and last HIV clinical stages increased the hazard of AIDS related to mortality. Additionally, male gender, older age, incarceration history, and last HIV clinical stages increased the non-AIDS mortality.

**Conclusions:**

Mortality caused by AIDS and non-AIDS remains high among people with HIV in Iran, particularly among males and those with late diagnosis. It seems that applying effective strategies to identify infected individuals at earlier stage of the infection, and targeting individuals with higher risk of mortality can decrease the mortality rate among HIV infected people.

## Background

Human Immunodeficiency Virus (HIV) is still a major public health issue in all around the world. Since the beginning of the AIDS epidemic, approximately 78 million people have been infected by HIV, and 35 million people have died because of AIDS-related diseases [1]. In the recent years, access to Highly Active Anti-Retroviral Therapy (HAART) has dramatically changed the AIDS epidemic, along with the rate of AIDS-Related Deaths (ARMs) [2, 3]. Therefore, ART is expected to help People Living With HIV (PLWH) to have the similar life expectancy to the general population [4, 5]. However, this goal has not been achieved, yet [6–8]. It has been predicted that increase in access to HAART and longer survival time would lead to more variations in the death causes among PLWH [3, 9, 10]. This is due to that as the proportion of ARMs is reducing because of HAART, PLWH are increasingly experiencing Non-AIDS-Related Deaths (NARMs) [11, 12].

HIV is also a public health challenge in Iran. According to the latest estimates of the United Nations Program on HIV/AIDS (UNAIDS), a total of 61,000 people (95% CI = (34,000, 120,000)) are living with HIV in Iran until 2018 [13]. Out of these patients, 9764 died and 14,656 of them entered the AIDS stage [14]. Based on the number of registered patients, Fars province has the second rank in the country with approximately 5000 registered HIV-infected patients. According to the UNAIDS report, 4000 (2500–6200) cases of ARM were recorded in Iran, and the rate of newly detected HIV-infected cases has increased about 21% until 2016. However, it appears that ARM events have reduced by 14% since 2010 [15]. This reduction might be attributed to the change in the treatment policy of the time of starting HAART for PLWH. In Iran, until 2018, HAART was to be started when CD4 count was reduced to < 500 cell/mm^2^ (200–350 cell/mm^2^ before 2015). However, since 2019, the treatment regimen has been changed and HAART must currently be started as soon as the individual is diagnosed with HIV infection regardless of the level of CD4 [16].

The importance of analysis of data on death among PLWH has been shown in several studies. The results of such studies could identify the strengths and weaknesses of health policies for treatment of HIV patients, to achieve a longer time of survival [17–19]. The 90–90-90 targets have been set to be reached by 2020. In this regard, 90% of all patients with HIV infection will know their disease statuses, and at least 90% of them will receive ART, and also 90% of them will suppress their viral loads with sustained treatment [20]. Clearly, defining the factors causing death among HIV patients can help move towards the targets [21].

Recent studies have indicated that the main causes of death among PLWH are co-infections, such as oncogenic viruses, Hepatitis B Virus (HBV), Hepatitis C Virus (HCV), tuberculosis, medication-related toxicities, and consumption of illegal or recreational drugs. Moreover, several cohort studies have demonstrated that HIV/AIDS-related conditions are the most frequent causes of death [2, 3]. However, the causes of mortality, including non-AIDS-related malignancies, cancers, liver and renal failures, cardiovascular diseases, and drug abuse are getting more diverse [10, 22, 23]. Despite the large number of studies conducted on the causes of death among PLWH, no efficient study has been performed in this field in Iran up to now. Therefore, new studies are required to determine the prognostic factors affecting the survival of PLWH in Iran.

In the present study, HIV patients’ survival was investigated among the patients who were registered in Shiraz HIV/AIDS Research Center during 1997–2017. With respect to the standard guidelines, the causes of death among HIV patients are classified to ARM and NARM [10, 11, 24]. To provide a valid method to simultaneously detect the risk factors of both types of events, a stepwise algorithm in the competing risks model was used [25].

## Methods

### Study setting

**Fig. 1:**
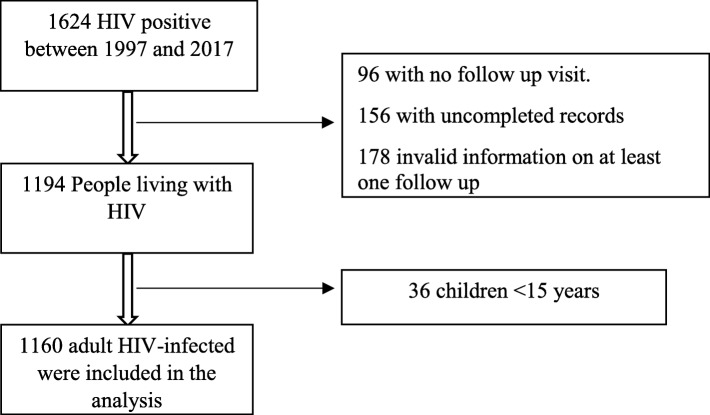
Profile of the study

This follow-up study was conducted at Shiraz HIV/AIDS Research Center, a referral center for individuals living with HIV in Fars province, Iran. Nearly 5000 patients were diagnosed at this center and almost 1160 cases followed their ART line with complete medical files. Data were recorder from all patients at this referral center from August 1997 to May 2017 and were extracted to a checklist. The checklist was completed by trained staff in terms of the World Health Organization’s (WHO) recommendations (Fig. 1).

### Data collection

The required data were extracted from the patients’ medical files by trained staff. The files included the patients’ socio-demographic and clinical data. Patients who had any special condition, such as pregnancy and the age under 16 years old, were excluded due to their very small numbers and special clinical conditions.

### Coding the cause of death

The response variable was defined as the time interval from HIV detection to death. For The individuals who were alive by May 2017, the times of data extracting, were considered as “censored”. ARM and NARM were classified based on the coding causes of death in HIV (CoDe) protocol [10]. The CoDe protocol is a uniform coding method that was developed through Copenhagen HIV Program (CHIP) (Copenhagen University, Copenhagen, Denmark) to collect data on the causes of death and its contributing factors. Deaths caused by AIDS or immunodeficiency conditions were classified as ARM. With respect to the CoDe protocol, ARM included three categories: opportunistic diseases, cancers such as cervical cancer, and other causes related to HIV/AIDS. On the other hand, NARM was defined as HIV-unrelated mortality, which included five categories: external causes (unexpected death like accident or overdose), hepatitis and liver-related deaths, Cardiovascular Diseases (CVD), cancers unrelated to HIV like lung cancer, and other causes. All deaths with unidentified causes were included in the analysis, if at least one CD4 was recorded in 6 months before the patient’s death. The individuals who died with CD4 counts < 200 cells/mm^2^ were classified as ARM, while those who died with higher CD4 counts were considered as NARM. Categorization of the causes of death was accomplished based on the recorded underlying causes, and was independently performed by two qualified physicians specialized in HIV/AIDS care. In case of disagreement, a third expert was involved.

### Variables

Two sets of covariates were considered. The first set of covariates was evaluated at diagnosis, which included age (continuous), gender (male, female), education level (less than secondary, secondary or above), marital status (married, unmarried, widowed/divorced), employment status (employed [i.e., reported having a job], unemployed), self-reported mode of HIV transmission (sexual, Injection Drug Use (IDU), etc.), and CD4 count (continuous). The other set of covariates was measured after diagnosis (i.e., in the follow-up period), which included drug abuse (none, drug used but being on Methadone Maintenance Therapy (MMT), drug use), linkage to HIV care defined as having visited centers providing HIV care and having access to ART and medical care after being diagnosed with HIV (yes, no), response to ART defined as indicating an improvement in clinical factors and/or increased CD4 counts by passing 6 months from ART initiation based on the medical records and physicians’ feedbacks (yes, no), late HIV diagnosis defined as less than 1 year between HIV diagnosis through an advanced phase of AIDS (yes, no), taking Pneumocystis Pneumonia (PCP) prophylaxis (yes, no), having HCV co-infection (yes, no), having HBV co-infection (yes, no), having tuberculosis co-infection (yes, no), and AIDS clinical stage defined based on WHO’s criteria (stages 1 and 2 vs. stages 3 and 4).

### Statistical analysis

Descriptive statistics were reported as mean, Standard Deviation (SD), median, and interquartile range (Q1, Q3) for continuous measures, and as absolute and relative frequencies for categorical ones. In order to accomplish survival analysis, Cox model and competing risks analysis method were used to apply Hazard Ratio (HR) and Sub-distribution Hazard Ratio (SHR) for both groups of death events as they were proposed by Fine and Gray (1999) [26]. At First, an adjusted competing risk and Cox regression model was run for the first set of covariates (i.e., those obtained at diagnosis). Then, a second set of forward stepwise competing risk and Cox regression model was run for the covariates measured at the follow-up. Additionally, these covariates were adjusted for those measured covariates at diagnosis with *p*-values less than 0.20. All data analyses were carried out using Stata, version 14.

## Results

In this study, a total of 1160 AIDS patients were enrolled due to the inclusion criteria. The mean age of the patients at diagnosis was 35.22 years old (SD = 8.43). Additionally, 1109 patients (95.6%) had not finished the compulsory education. Besides, the majority of them were male (*n* = 857, 73.9%). The transmission route was mainly injecting drugs in males (*n* = 719, 83.9%) and sexual contact in females (*n* = 262, 86.5%). All descriptive statistics of the study variables have been presented in Table 1. Totally, 391 patients (33.7%) died during the study period. Regarding, among these cases, 251 were considered as ARM (64.2%) and 140 of them were considered as NARM (35.8%). Opportunistic and AIDS-defining infections (*n* = 241, 96%) were accounted for the main causes of ARM. Among these cases with NARM, the most frequent cause of death was external causes (*n* = 78, 55.7%), including overdoses and driving accidents. Moreover, 14 deaths (7%) were related to cancer as shown in Table 2.

**Table 1:** Characteristics of individuals living with HIV at diagnosis and follow up (total *N* = 1160)

Baseline Characteristics	Entire cohort	AIDS-related mortality	Non-AIDS-related mortality	All-cause mortality
Overall	1160 (100)^*^	251 (21.6)	140 (12.1)	391 (33.7)
Gender				
Men	857 (73.9)	215 (25.1)	136 (15.9)	351 (41.0)
Women	303 (26.1)	36 (11.9)	4 (1.3)	40 (13.2)
Age				
Median (Q1, Q3)	34 (29–40)	36 (30–43)	35 (30–41)	35 (30–42)
< 30	309 (26.6)	55 (17.8)	34 (11.0)	89 (28.8)
30–39	545 (47.0)	108 (19.8)	61 (11.2)	169 (31.0)
> =40	306 (26.4)	88 (28.8)	45 (14.7)	133 (43.5)
Marital status				
Married	526 (45.3)	101 (19.2)	48 (9.1)	149 (28.3)
Single	303 (26.2)	81(26.7)	50 (16.5)	131 (43.2)
Widowed/divorced	331 (28.5)	69 (20.8)	42 (12.7)	111 (3.4)
Education				
Less than secondary	403 (34.7)	93 (23.0)	50 (12.4)	143 (35.5)
Secondary or more	757 (65.3)	158 (20.8)	90 (11.8)	237 (31.3)
Employment				
Employed	549 (47.3)	112 (20.4)	77 (14.0)	189 (34.4)
Unemployed	611 (52.7)	139 (22.7)	63 (10.3)	202 (33.1)
Incarceration history				
No	392 (33.8)	48 (12.2)	10 (2.6)	58 (14.8)
Yes	768 (66.2)	203 (26.4)	130 (16.9)	333 (43.4)
Mode of HIV transmission				
Sexual	344 (29.7)	46 (13.4)	9 (2.6)	55 (16.0)
Injection drug use	733 (63.2)	185(25.2)	123 (16.8)	308 (42.0)
Others^a^	83 (7.1)	20 (24.1)	8 (9.6)	28(33.7)
CD4 count Median (Q1, Q3)	215 (102,352)	238 (127,254)	133 (63,260)	163 (78,300)
Year HIV was diagnosed				
Before 2011	712 (61.4)	46 (6.4)	35 (4.9)	81 (11.3)
2011–2014	306 (26.4)	145 (47.3)	69 (22.5)	214 (69.8)
2015–2017	142 (12.2)	60 (42.2)	36 (25.3)	96(67.5)
Covariates measured at follow-up				
Drug use status				
No drug use	307 (26.5)	36 (11.7)	5 (1.6)	41 (16.0)
On methadone therapy	568 (49.0)	101 (17.8)	65 (11.4)	166 (42.0)
Drug users	258 (24.5)	114 (44.2)	70 (27.1)	184 (33.7)
Linkage to HIV care				
No	219 (18.9)	124 (56.6)	72 (32.9)	196 (89.5)
Yes	941(81.1)	127 (13.5)	68 (7.2)	195 (20.7)
Responded HAART 6 month after initiation^b^				
No	392 (33.8)	239 (61)	97 (24.7)	232 (59.2)
Yes	768 (66.2)	12 (1.6)	43 (5.6)	159 (20.7)
Late HIV diagnosis				
No	629 (54.2)	136 (21.6)	96 (15.3)	232 (36.9)
Yes	531 (45.8)	115 (21.7)	44 (8.3)	159 (29.9)
TB status				
Negative	1048 (90.3)	205 (19.6)	123 (11.7)	328 (31.3)
Positive	112 (9.7)	46 (41.0)	17 (15.2)	63 (56.3)
PCP prophylaxis				
No	480 (41.4)	108 (22.5)	77 (16.0)	185 (38.5)
Yes	680 (58.6)	143 (21)	63 (9.3)	206 (30.3)
HCV positive co-infection				
Negative	500 (43.1)	116 (23.2)	33 (6.6)	149 (29.8)
Positive	660 (56.9)	135 (20.5)	107 (16.2)	242 (36.7)
HBV positive co-infection				
Negative	1078 (92.9)	231 (21.4)	127 (11.8)	358 (33.2)
Positive	82 (7.1)	20 (24.4)	13 (15.9)	33 (40.2)
Last clinical stage				
1,2	832 (71.7)	24 (2.9)	91 (10.9)	115 (13.8)
3,4	328 (28.3)	227 (69.2)	49 (14.9)	276 (84.1)

* Data are presented as N (%)

^a^Other modes of transmission included: who infected HIV with unknown cause, blood transition or through dentistry

^b^include those showing an improvement in clinical factors or increased CD4 counts 6 months after HAART treatment based on medical records and physicians’ comments

**Table 2:** Death category of individuals living with HIV

	Non-AIDS-Related Death (NARD)					AIDS-Related Death (ARD)		
	Cancer^c^	Other diseases^b^	CVD	Hepatic/liver-related	External death^a^	Other diseases^b^	Cancer^c^	Opportunistic
Number of deaths	6 (4%)	10 (8%)	12 (9%)	34 (24%)	78 (55%)	2 (1%)	8 (3%)	241 (96%)

^a^External death: Sudden death, including accident, overdose, and suicide

^b^Other diseases: Unknown death categorized based on CD4 count within 6 months prior to the date of death. The CD4 cell count of lower than 200 cells/mm^2^ was categorized as ARM and otherwise as NARM

^c^Cancer: Based on the CoDe protocol, AIDS-related cancers like cervical cancer were considered as ARM and other causes unrelated to HIV like lung cancer were categorized as NARD

**Fig. 2:**
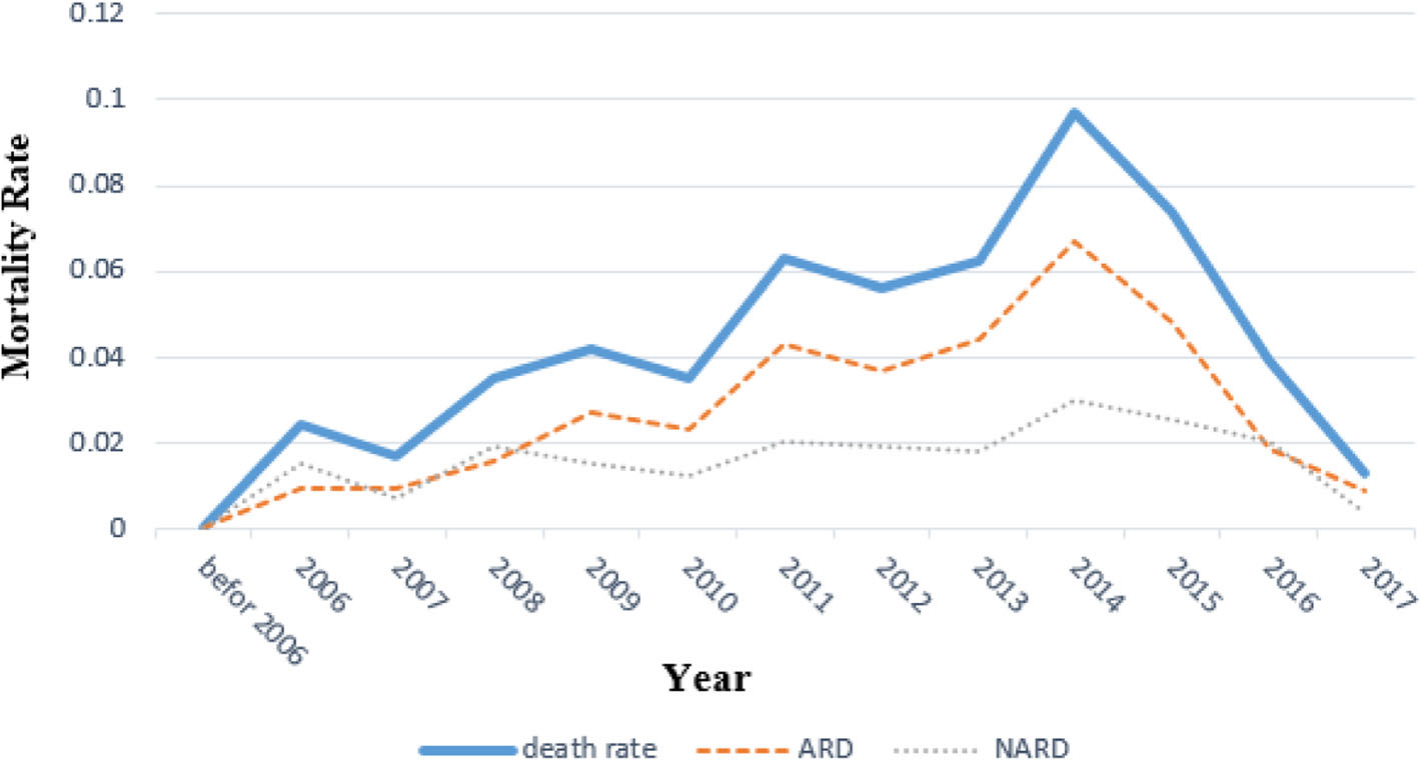
Mortality rates in different years for total, AIDS-related and non-AIDS-related death in AIDS patients

**Fig. 3:**
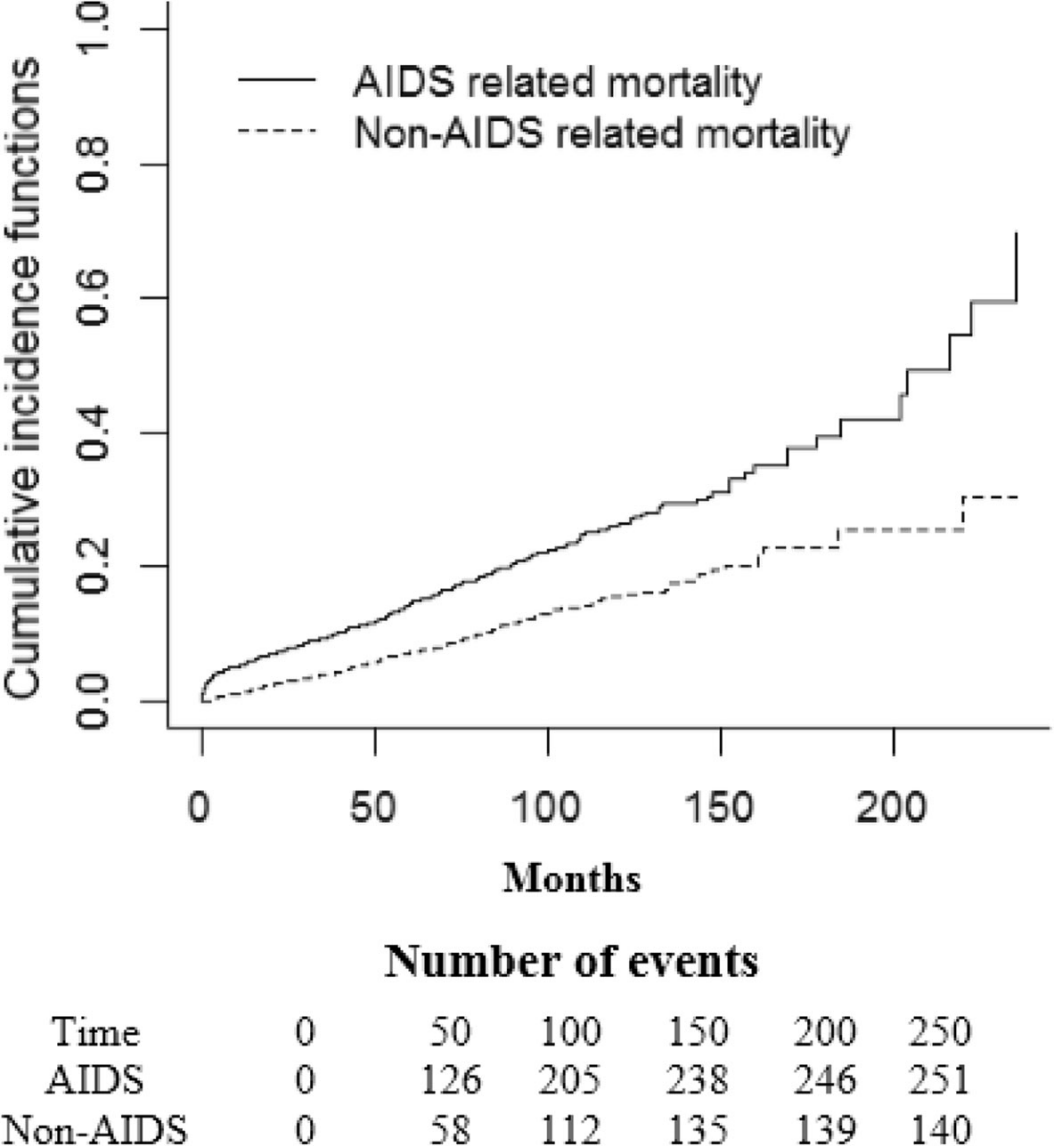
Cumulative incidence function for AIDS-related death and non-AIDS-related death

The results revealed different trends of mortality for different causes of death. The total rate of mortality increased with a starting rate as two deaths/100 person-years in 2006 and raised to 10 deaths/100 person-years in 2014. Afterwards, a remarkable fall was detected in 2016 (one death/100 person-years). As most causes of death were AIDS related, ARMs had a similar trend to all-cause mortality. However, NARM remained fairly stable with a slight increase at the end of the study (from approximately one death/100 person-years to two deaths/100 person-years) (Fig. 2). Moreover, the cumulative incidence of ARM was more in comparison with that of NARM during the study (Fig. 3).

Based on the results of survival analysis on baseline characteristics, two variables; i.e., education level and employment status, had *p*-values more than 0.2. Therefore, they were excluded from all three multivariate models (Table 3). Moreover, mode of HIV transmission were not significant for the ARM model and CD4 count for the NARM model (*p* > 0.2) and consequently were removed.

**Table 3:** Mortality rates and adjusted hazard ratio baseline characteristics from Cox and competing risk model on AIDS-related, non- AIDS-related and all-cause mortality among people living with HIV (*N* = 1160)

Baseline Characteristics	AIDS-related mortality		None-AIDS		All-cause mortality	
	Mortality rate (1000 person month) (95% CI)	Adjusted HR (95% CI)	Mortality rate (1000 person month) (95% CI)	Adjusted HR (95% CI)	Mortality rate (1000 person month) (95% CI)	Adjusted HR (95% CI)
Overall	3.2(2.9,3.7)	–			4.5(4.1,5)	–
Gender						
Men	3.8(3.3,4.3)	1.09(0.57,2.08)	2.1(1.7,2.4)	3.82(1.29,11.36)	5.3(4.8,5.9)	1.38(0.81,2.33)
Women	1.8(1.3,2.5)				2(1.5,2.7)	
Age						
< 30	2.2(1.7,2.8)				3.2(2.6,3.9)	
30–39	3(2.5,3.6)	1.29(0.94,1.77)	1.5(1.2,2)	1.25(0.83,1.9)	4.3(3.7,5)	1.35(1.04,1.75)
> =40	5.4(4.4,6.7)	2.08(1.48,2.93)	2.4(1.8,3.2)	1.82(1.15,2.87)	7.1(6,8.4)	2.36(1.79,3.12)
Marital status						
Married	2.8(2.3,3.4)				3.8(3.2,4.5)	
Single	4.3(3.4,5.3)	1.33(0.98,1.81)	2.3(1.7,3)	1.43(0.96,2.15)	6(5,7.1)	1.46(1.15,1.87)
Widowed/divorced	3.1(2.5,4)	0.99(0.72,1.37)	1.7(1.2,2.3)	1.45(0.95,2.2)	4.4(3.7,5.3)	1.13(0.88,1.46)
Education						
< Secondary	3.4(2.8,4.2)				4.6(3.9,5.5)	
> = Secondary	3.2(2.7,3.7)	0.99(0.76,1.28)	1.6(1.3,2)	1(0.71,1.4)	4.5(3.9,5.1)	1.01(0.82,1.24)
Employment						
Employed	2.9(2.4,3.5)				4.4(3.8,5)	
Unemployed	3.6(3,4.2)	1.29(1.05,1.59)	1.5(1.1,1.9)	1.06(0.76,1.48)	4.7(4.1,5.4)	1.29(1.05,1.59)
Incarceration history						
No	1.8(1.4,2.4)				2.2(1.7,2.8)	
Yes	4(3.4,4.5)	1.69(0.97,2.94)	2.2(1.8,2.6)	2.01(0.92,4.35)	5.6(5,6.2)	1.85(1.19,2.85)
Mode of HIV transmission						
Sexual	2(1.5,2.7)				2.4(1.8,3.1)	
Injection drug use	3.7(3.2,4.3)	0.89(0.52,1.53)	2.1(1.8,2.5)	1.64(0.79,3.41)	5.3(4.8,6)	1.12(0.74,1.69)
Others	4(2.6,6.2)	1.3(0.68,2.48)	1.5(0.7,3)	1.76(0.67,4.58)	5.2(3.6,7.5)	1.56(0.94,2.58)
CD4 count						
< 200	4.6(4,5.4)	4.81(2.28,10.15)	1.7(1.4,2.2)	1.1(0.75,1.62)	6.4(5.6,7.3)	2.34(1.46,3.74)
200–350	2.2(1.7,2.8)	2.48(1.15,5.35)	1.6(1.2,2.1)	1.18(0.74,1.9)	3.8(3.1,4.6)	1.48(0.91,2.43)
350–500	1.3(0.8,2)	1.53(0.66,3.55)	1.5(1,2.3)	1.3(0.7,2.44)	2.8(2.1,3.8)	1.18(0.69,2.03)
> 500	0.8(0.4,1.7)					

The results of forward stepwise model proposed that “response to treatment” and “PCP prophylaxis” were strong protecting factors for all-cause mortality, ARM, and NARM. Accordingly, “response to treatment” declined the all-cause mortality by 95% (HR = 0.05; *p* < 0.001). Actually, based on the “response to treatment”, ARM and NARM hazards were reduced by 91% (SHR = 0.09; *p* < 0.001) and 86% (SHR = 0.14; *p* < 0.001), respectively. Similarly, “PCP prophylaxis” reduced all-cause mortality by 57% (HR = 0.43; *p* < 0.001), ARM by 67% (SHR = 0.33; *p* < 0.001), and NARM by 36% (SHR = 0.64; *p* = 0.021). Also, “Last clinical stage” was significantly associated with both ARM and NARD; however, it has no relationship with all-cause mortality. In addition, “stages 3 and 4” were associated with a higher risk of ARM (SHR = 7.4; *p* = 0.025) and a lower risk of NARM (SHR = 0.24; *p* < 0.001). Late HIV diagnosis, age, and CD4 count at diagnosis were identified as significant risk factors for ARM and all-cause mortality, but not for NARM. Each unit increase in baseline CD4 count declined all-cause mortality by 0.1% (HR = 0.999; *p* < 0.001) and ARM by 0.2% (SHR = 0.998; *p* < 0.001). Additionally, 1 year increase in age of diagnosis, caused a 4% increase in all-cause mortality (HR = 1.04; *p* < 0.001) and a 2% increase in ARM risk (SHR = 1.02; *p* < 0.001). Moreover, “late HIV diagnosis” was the most effective risk factor because it increased all-cause mortality and ARM risk by four and about three folds, respectively (HR = 4.05; *p* < 0.001) (SHR = 2.84; *p* < 0.001). Furthermore, incarceration history increased the all-cause mortality by about 70% (HR = 1.69; *p* = 0.005), and the risk of NARM by two folds (SHR = 2.14; *p* = 0.007). Finally, gender (SHR = 3.57; *p* = 0.027) was a significant risk factor only for NARM (Table 4).

**Table 4:** Forward stepwise Cox and competing risk model on AIDS-related, non- AIDS-related and all-cause mortality among people living with HIV (*N* = 1160)

	*AIDS-related mortality*	*Non-AIDS-related mortality*	*All-cause mortality*
Characteristics at diagnosis	Adjusted SHR (95% CI)	Adjusted SHR (95% CI)	Adjusted HR (95% CI)
Gender (men vs. women)		3.57 (1.16,11)	
Increased age	1.02 (1.00,1.04)		1.04 (1.02,1.05)
Incarceration history		2.14 (1.04,4.41)	1.69 (1.26,2.26)
Increased CD4 count	0.998 (0.997,0.999)		0.999 (0.998,0.999)
Covariates measured at follow-up			
Responded HAART 6 months after initiation	0.09 (0.04,0.20)	0.14 (0.09,0.23)	0.05 (0.04,0.07)
Late HIV diagnosis	2.84 (2.12,3.80)		4.05 (3.20,5.12)
Receiving PCP prophylaxis	0.33 (0.22,0.51)	0.64 (0.46,0.89)	0.43 (0.35,0.54)
HIV Clinical stage (3,4 vs. 1,2)	7.40 (3.89,14.07)	0.24 )0.15,0.38)	

## Discussion

The results of this study demonstrated that not only ARM is still a major cause of death, but also NARM poses a threat to PLWH. This is in agreement with other studies, as well [10, 27]. It seems that after ART, no effective policy has been taken to control NARM in HIV patients in Iran. In contrast to other studies, in which non-AIDS-related malignancies at older ages were the main reason for NARM [2, 3, 28], among Iranian HIV patients, sudden death was more common. External death comprised about half of NARMs (*n* = 77, 55% of NARMs). Accordingly, overdose most frequently caused NARMs. However, most NARMs originated from non-AIDS malignancies, CVD, and liver diseases in developed countries. Indeed, ART has been reported to be more effective in developed countries where HIV patients receiving ART were shown to survive as long as the normal population do. As a result, the mean age in NARMs can be higher than that in ARMs [29, 30]. However, like a study in South Africa [31], the present study findings indicated that most NARM events were related to overdose and addiction, which occurred at the initial stages of HIV infection and lower ages. In this study, the majority of NARMs were from male patients whose their route of HIV transmission was drug injection, and also the cause of their death was overdose (*n* = 56, 71% of external deaths). In fact, drug abuse has reduced the survival of HIV patients through raising NARMs even prior to the AIDS stage. In consistent with that, a recent study showed an association between serum albumin and NARM events and suggested that the association was stronger for smokers [32].

Previous studies indicated a rapidly increasing number of drug users in Iran from 1998 to 2003 [33–35]. This could have led to higher HIV infection and larger number of deaths in the following years. According to the findings of the current study, the mortality rate reached its peak in 2014 (Fig. 2). On the other hand, case finding of HIV infection has become more efficient in the recent years. Consequently, the number of registered HIV patients has increased and medical care is going to be started earlier for HIV patients, along with reducing the death rate. In 2016, the rate of ARM decreased and became almost equal to that of NARM. These findings were consistent with those of other studies conducted in different countries [2, 3, 28].

In the present study, almost 34% of the patients died during the study period, and about two out of every three deaths were related to opportunistic and AIDS-defining infections with a history of addiction. These findings indicated that addiction had a significant effect on the causes of death and survival of HIV patients in south of Iran regions. On the contrary, several studies from other countries have reported other factors as the major causes of death [2, 3, 28]. More recently, Weber et al. disclosed that non-AIDS malignancies, HIV/HCV-co-infection, liver failure, non-AIDS infections, substance use, and suicide were the main causes of NARM in Switzerland [3]. Morlat et al. also indicated that the most frequent NARM events in France were non-viral hepatitis-related and liver-related malignancies, CVD, and non-AIDS-related infections [11].

Based on the current study findings, late HIV diagnosis in older ages, in stages 3 or 4, or with a lower CD4 count could significantly increase the risk of ARM. These results were in line with the findings of other studies [36, 37]. The results regarding NARM indicated that the male patients in earlier stages and those who had a history of incarceration, also had a significantly shorter survival time. These results were not in agreement with those reported from several developed countries [28]. In a study on low-income individuals in Brazil, Albuquerque et al. claimed that tuberculosis, anemia, and CD4 count lower than 200 cell/mm^2^ were the main reasons for NARM [22].

The present study findings demonstrated a significant improvement in detection and treatment of HIV patients. However, the 90–90-90 targets have not been fulfilled in Iran [21]. In 2010, Prasada et al. predicted that control of HIV epidemics might be unsuccessful in Asia. Therefore, there is a prompt need determining sustainable prevention and treatment strategies and concentrating on programs that are highly effective in preventing the disease, as well as decreasing the number of new HIV infections [38].

The strengths of the present study were using a relatively large sample and making use of competing risks model for performing statistical analysis of the data. However, limitation of this investigation were incomplete medical files, especially about viral load check before 2011. Another study limitation was the restricted access to the patients’ information, especially for the decreased ones. Another important limitation of this study was that, like several other diseases [39, 40], the detection rate of HIV/AIDS is not conveniently high in Iran due to such reasons as the stigmatized attitude of the population and the healthcare personnel [41, 42]. Moreover, we were not able to include one of the HIV exposure categories, MSM (i.e., men who have sex with men) due to its unavailability and that such data are not usually collected in the routine surveillance system in Iran. However, this might not be an issue as the two routes of injection drug use and heterosexual contacts have been considered as the main HIV transmission routes in Iran.

## Conclusion

The results of the present study indicated that both ARM and NARM are substantially high among people with HIV in Iran. Applying effective strategies is needed to achieve on time diagnosis of individuals with HIV and provide them with HIV care and treatment services to enhance the survival of the patients. Moreover, males living with HIV require more attention when receiving HIV care and treatment as they experience a higher risk of NARM. In line with the available evidence, the findings of the current research revealed that early HIV care and treatment could substantially reduce ARM and NARM among HIV patients. Therefore, to reduce mortality rate among people with HIV, the current strategies should be revised to improve the timing of treatment initiation and also to optimize the adherence to the treatment.

## Data Availability

The data that support the findings of the present study are available from the Iran’s Ministry of Health (MOH) and are not publicly available. The anonymized dataset used for the present research is however available from the corresponding author on reasonable request and with permission of both Ministry of Health and Shiraz HIV/AIDS Research Center.

## Abbreviations

AIDS: Acquired Immune Deficiency Syndrome
ARD: AIDS-Related Death
HIV: Human Immunodeficiency Virus
NARD: Non-AIDS-Related Death
PLWH: People Living With HIV
SHR: Sub-distribution Hazard Ratio
